# 

**Published:** 1782

**Authors:** 


### THE LONDON MEDICAL JOURNAL.

### VOLUME THE THIRD.

Jan. Feb. & Mch. 1782

Surgeon Genl's Office
LIBRARY
Washington, D.C.
63442
S.G.O.

LONDON:

Printed for the EDITOR by W. RICHARDSON,

AND SOLD

By JOSEPH JOHNSON, N° 72, St. Paul's Church Yard.

M DCC LXXXIII.

### PROMOTED.

J. S. Hausmann, to be professor of anatomy at Brunswick in the room of the late Dr. Rollin. —M. Barthez, professor of physic at Montpellier, to be first physician to the Duke of Orleans at Paris in the room of the late Dr. Tronchin.—J. C. F. Voitus to be professor of surgery in the Royal medico-chirurgical college at Berlin.—Christ. Fred: Nurnberger, M. D. to be professor extra. of physic at Wittemberg.—J. C. Starke, to be professor extra. of physic at Jena.—Sam. Thomas Soemmering, M.D. to be professor of anatomy and surgery at Cassel.—John Godfrey Leonhardi to be professor extra. of physic at Leipsic.—Dr. William Hunter to be one of the eight foreign members of the Royal academy of sciences at Paris, in the room of the late Dr. Tronchin.—Dr. William Cleghorn, to be lecturer in anatomy in Trinity-college, Dublin, in the room of his uncle, Dr. George Cleghorn, who resigned in his favour.

1782, *January* 9. Dr. John Cooke to be physician to the General Dispensary in London, in the room of Dr. Nathaniel Hulme, who has resigned.—23. Dr. John Turton to be physician extra. to the King, and physician in ordinary to the Queen, in the room of the late Sir J. Pringle, Bart.—Dr. Thomas Gisborne to be physician to the Queen's houshold in the room of Dr. Turton.—26. Mr. Robert Barnewall to be apothecary to the General Hospital in the Leeward islands.

*Feb.* 9. Mr. John Stewart to be surgeon to the Royal regiment of horse-guards, in the room of James Mackenzie, M. D.—16. Mr. William Turner, hospital mate, to be surgeon to the 104th regiment of foot.—Mr. Home to be surgeon in ordinary to the Savoy prison.

---

### D I E D.

1780, *March* 23. At St. Petersburgh, John James Lerche, first physician to the Russian army; born at Potsdam in 1703.

*July* 23, at Dresden, Charles Gesner, M. D. physician to the Elector of Saxony.

*Sept.* 27, at Hall in Saxony, Dr. Adam Niesky, professor of physic.

1781. At Paris, Julian Buffon, M. D. first physician to the Countess d'Artois, and editor of the French translation of Dr. James's Medical Dictionary.—In the same city, Anthony Casamajors, M. D.—At Brunswick, aged 74, Ernest Jeremiah Rollin, M. D. professor of anatomy.

*Jan.* 1. Of apoplexy at Rome, aged 68, John Lewis Bianconi, M. D. minister from the Court of Saxony to that of Rome, and one of the foreign members of the Royal Academy of surgery at Paris.—Some time in *January*, at Bremen, aged 55, J. G. Runge, M. D. professor of anatomy and natural philosophy.

*Feb.* 21. At Gahard, near Rennes in the province of Brittany, in France, Joseph Exupere Bertin, M. D. member of the faculty of physic, and of the Academy of Sciences at Paris, author of an ingenious Treatise on Osteology, in 4 vols. 12mo. published at Paris in 1754, and of several anatomical papers in the Memoirs of the Academy of Sciences. He was born at Trenlay in the diocese of Rennes, June 25, 1712. In 1744, he was appointed by the faculty joint professor of midwifery with the late M. Astruc. The same year he was seized with a sina ſingular complaint. It began with a delirium which, after continuing for three days, terminated in a ſtate of lethargy. When he was ſufficiently recovered from this attack, he tried the effects of his native air. He experienced ſeveral relapſes, but in 1750 perfectly recovered his health and intellectual faculties, and returned to Paris, where he married in 1765. After the death of his wife he retired to Gahard. He has left behind him a large work on the arteries, begun in 1739, and preſented to the Academy of Sciences in 1775, for their approbation. This work is expected ſoon to be publiſhed.—

*Dec.* 15. At Forfar in Scotland, the Rev. J. Carr, M. D.

1782. *Jan.* 7. At Glaſgow, Mr. Thomas Hamilton, Profeſſor of Anatomy and Botany in the Univerſity at that place.—13. At Preſcot, in Lancaſhire, Mr. David Houghton, Surgeon and Apothecary.——15. At Dublin, William Clement, M. D. Regius Profeſſor of Phyſic, Vice Provoſt of Trinity College, and Member of Parliament for that city.—17. In Bolt-court, Fleet-ſtreet, at the houſe of his friendly patron Dr. Samuel Johnſon, Mr. Robert Levet, aged 80, a uſeful and charitable practitioner of Phy-

ſic; fic; born near Hull, in Yorkfhire.—19. About ten at night in St. Alban's-ftreet, Weftminfter, aged 78, Sir John Pringle, Bart. phyfician extra to the King, phyfician to the Queen, late Prefident of the Royal Society, &c. His life, written by the Rev. Dr. Kippis, is now in the prefs, fo that we hope foon to be enabled to prefent our readers with authentic memoirs of this learned and illuftrious phyfician. —Same day, at Carlifle, Mr. Jacob Coulthard, formerly a furgeon and apothecary at Wigton. —22. At Derby, aged 24, Mr. Ferdinand Brown, furgeon and apothecary.——30. At Mucairn in Argylefhire, aged 101, Mr. Archibald M'Calman, furgeon. He made the laft 35 years of his life, a period of hermetifm, devoted to religion. He ftudied under Dr. Pitcairn, at Edinburgh, the firft and fecond years of the prefent century. Born in Charles the fecond's time, he lived to fee the twenty-fecond year of the feventh reign in his days. During this fpace, four civil wars at thirty years diftance from each other difturbed government.

*Feb.* 4. At Glafgow, aged 82, Mr. David Paten, fenior member of the faculty in that city.

city.—9. At Tunbridge in Kent, Mr. Thomas Slatter, surgeon and apothecary, and member of the Medical Society at Edinburgh.—Same day, at Salford, in Lancashire, Mr. Thomas Walton, apothecary.——14. At Rochdale, in Lancashire, Mr. Cable, apothecary.—22. At Bath, Mr. Gye, apothecary.—28. At Little Chelsea, Mr. John Morton, surgeon in ordinary to the Savoy Prison.

*March* 13. At Richmond, Surry, aged 82, John Baker, M. D.

---

### SECTION IV.

### QUARTERLY CATALOGUE.

1. A Letter to Dr. Robert Jones of Caermarthenshire, in answer to the account which he has published of the case of Mr. John Braham Isaacson, student of medicine, and to the injurious aspersions which he has thrown out against the physicians who attended Mr. Isaacson. By *Andrew Duncan*, M. D. Fellow of the Royal College of Physicians, Edinburgh; and member of the Royal Societies of Medicine of Paris, Copenhagen

Copenhagen, Edinburgh, &c. 8vo. *Elliot*, Edinburgh; and *Dilly*, London, 1782. 46 pages. 1s.

We have not yet ſeen the pamphlet to which this is an anſwer, but the plain narrative of facts contained in the publication before us, confirms us in the high opinion we have long entertained of the abilities and integrity of the learned author, and convinces us that his reputation has been very illiberally and unjuſtifiably attacked. It ſeems, however, that Dr. Jones in his *Inquiry into the State of Medicine* has not confined his ſtrictures ſolely to Dr. Duncan, as ſeveral other reſpectable characters, particularly, Dr. Cullen, Dr. Monro and Mr. Alex. Wood are ſaid to come in for a ſhare of abuſe.

Mr. Iſaacſon, the young gentleman whoſe caſe is the principal ſubject of the preſent pamphlet, was attacked, we are told, in October, 1780, with a diſtinctly marked fever, attended with an evident diſpoſition to the putrid ſtate. He was attended by our author and Dr. Monro. In the courſe of the diſeaſe, the patient had much delirium, and the ſymptoms of putreſcency ran to a great height, but by the uſe of proper remedies he recovered. Mr. Iſaacſon was no ſooner free from the ſymptoms of fever than than it began to be reported that his cure was owing to liberal doſes of rum and laudanum recommended by Dr. Brown, and conveyed to him by Dr. Jones. This pretended cure, it ſeems, is particularly inſiſted on in the *inquiry*, &c. publiſhed by the latter. As this ſtory made no little noiſe, and it was even confidently aſſerted, that without the interference of Dr. Brown, the patient would have died, our author thought it his duty to inquire into the matter. He found that the patient himſelf was entirely ignorant of the affair, but his nurſe and his landlady upon being interrogated, declared that during Mr. Iſaacſon's illneſs, and while he was delirious, Dr. Jones had repeatedly been to viſit him, and had endeavoured by intreaties, and even by the offer of a pecuniary reward, to prevail upon them to give him a mixture of rum and laudanum privately, without the knowledge of his phyſicians; and that at length in order to get rid of Dr. Jones's importunities they had ſeemingly acquieſced, but that the moment he left the room they threw the mixture into the aſhes. Dr. Duncan has inſerted at full length the depoſition of theſe two women, and likewiſe letters from Dr. Monro, Mr. Goodwin,

(a ſtudent

(a student of phyfic who lodged in the next room to the patient, and was prefent at every confultation of the two phyficians) and the patient himfelf who now refides at Landwade in the neighbourhood of Newmarket. Thefe letters give additional weight to the author's narrative by confirming the truth of every part of it in which either of them was concerned.

The pamphlet concludes with fome fevere ftrictures on the conduct of Dr. Jones and Dr. Brown.

2. A Narrative of a fingular Gouty Cafe; with obfervations. By *John Lee*, M. D. Phyfician at Bath, member of the College of Phyficians in London, and Fellow of the Royal Society. 8vo. *T. Evans*, London, 1782. 27 pages. 1s.

We have here the cafe of a patient who in his fixty-eighth year, after having been for twenty years fubject to frequent returns of the gout, was fuddenly feized with a violent ftrangury, accompanied with a fever: thefe fymptoms were removed in about a week, and foon after his recovery he obferved, every morning in the urine, which he had made the preceding night, a great quantity of fediment, of the confiftence of of glue diffolved in water. This fediment was of a pea green colour, and emitted a very offenfive fmell. The quantity of it was generally a half pint in three pints of urine. From that time till his death, whenever this humour was fuppreffed or even diminifhed, he laboured under a lownefs of fpirits, lofs of appetite, fwelled legs or the gout, and he ufed to fay that all thefe fymptoms proceeded from the fediments having gone aftray (as he termined it) for as foon as it began to return his complaints leffened, and he recovered his ufual ftate of health.—In the year 1777 this fediment was fuddenly fuppreffed by his walking in marfhy ground, and he was foon afterwards troubled with a fwelling of the right tefticle, and a fmart fever. Thefe fymptoms were relieved by a return of the flimy appearance in the urine. At length in March 1781, being then in his 77th year, fome irregularity in his mode of living was fucceded by a fevere cold, and brought on violent pains in his bowels, together with head ach and fever. The fediment, which had fo often relieved him by its return, was now fuppreffed for ever, and he died on the eleventh day of the diforder.

His body was examined after death, and in the bladder were found about two ſpoonfuls of the ſame kind of ſediment which had ſo often been obſerved in his urine. One ſide of the neck of the bladder appeared ſlightly inflamed, and the proſtate gland was ſomewhat enlarged.

3. Memoire pour M. D'Oſtertag, M. D. et accoucheur juré á Straſbourg, *i. e.* memorial in favour of M. Oſtertag, M. D. ſworn accoucheur at Straſburgh. 4to. Straſburgh, 1781. 39 pages.

A famous empirie, who ſtiles himſelf the Count de Caglioſtro, has lately made his appearance at Straſburgh. In the paper before us we are told that he employed the ſame remedies for an hundred different diſeaſes, and that a great number of perſons are ſaid to have fallen victims to his ignorance. Amongſt other patients he was deſired to viſit a poor woman in labour who had been under the care of our author. The Count adminiſtered a few drops of ſome elixir, as he called it, and the poor woman happening to be delivered ſoon after, this event was imputed to the medicine and Dr. Oſtertag experienced a great deal of calumny. 'Tis with a view to vindicate dicate himſelf from ſuch aſperſion that he publiſhes this memorial.

4 Diſſertatio Inauguralis medica exhibens de morbis nervorum obſervationes quædam ſingulares. Auctore *F. I. Bruckmann*, Brunſvicenſi 4to. Gotting 1780, p. 40.

The author relates four caſes found among the papers of his father who was phyſician to the duke of Brunſwick. The firſt is a caſe of St. Vitus's dance, brought on in a young girl, by a fright, and cured by the bark. The ſecond is a caſe of rheumatiſm in which the nervous ſyſtem was ſingularly affected. The third relates to a girl who was ſubject to the gout, and became ſo irritable as to have every kind of ſpaſm. In the fourth we have the hiſtory of a nervous diſorder brought on by the repulſion of a cutaneous eruption.

4. Experiences ſue les vegetaux, &c. *i. e.* Experiments on vegetables, By *John Ingenhonſz*, Phyſician to their Imperial Majeſties, &c. tranſlated from the Engliſh by the author. 8vo. Paris, 1780. 400 pages with plates.

5. Diſſertation chimique ſue les Eaux minerales de Lorraine, &c. *i. e.* A chemical diſſertation on the mineral waters of Lorraine, a work for for which the author received the prize offered by the academy of Nancy. By M. Nicolas, demonftrator of chemiftry at Nancy. 8vo. Paris, 1780. 116 pages.

6. Traité des Eaux minerales de Chateldon, de celles de Vichy et de Hauterive en Bourbonnois; avec le detail de leurs propriètés medicinales et leur analyfe. *i. e.* A treatife on the mineral waters of Chateldon, and of thofe of Vichy and Hauterive in the province of Bourbonnois; with an account of their medicinal properties, and their analyfis. By *M. Debreft*, M. D. &c. 12mo. Moulins, 1779. 359 pages.

7. Effai fur les alimens, pour fervir de commentaire aux livres dieteriques d'Hippocrate. *i. e.* An effay on aliments, intended as a commentary on the dietetic books of Hippocretes. A new edition corrected and enlarged. By C. *Lorry*, M. D. &c. 12mo. Paris, 1781. 2 vols.

8. Traité des propriétés et ufage de la douce amere, ou folanum fcandens, dans le traitement de plufieurs maladies, et furtout des maladies dartreuses. *i. e.* A treatife on the properties and ufe of the folanum dulcamara, in the treatment of feveral difeafes and particularly cutaneous eruptions.

eruptions. By *M. Carrere*, Regius Professor of physic, Censor Royal, formerly inspector of the mineral waters of Roussillon, &c. 8vo. Paris, 1781. 170 pages.

9. Memoires sur les observations meteorologiques faites á Franeker en Frise, pendant l'année 1779. *i. e.* Essays relative to the meteorological observations made at Franeker in Friesland, 1779. By *M. Van Swinden*, Professor of Philosophy in the University of Franeker. 8vo. Amsterdam, 1780. 336 pages.

10. Parere Medico-legale sopra un fetoa, &c. *i.e.* a medico-legal consultation concerning a fœtus that came into the world after a pregnancy of 196 days. By Lewis Bessi, M. D. 4to. Florence, 1780. 16 pages.

11. Traité generale des peches, et histoire des poissons ou des animaux qui vivent dans l'eau. *i. e.* a general treatise on fisheries, with a history of fishes or of animals that live in water. By *M. Duhamel du Monceau*, of the Academy of Sciences. Vol. III. sect. IX. 4to. Paris, 1781, with 27 copper-plates.

